# Fruticuline A, a chemically-defined diterpene, exerts antineoplastic effects in vitro and in vivo by multiple mechanisms

**DOI:** 10.1038/s41598-020-73432-2

**Published:** 2020-10-05

**Authors:** Claudia Rita Corso, Maria Carolina Stipp, Débora Rasec Radulski, Marihá Mariott, Luisa Mota da Silva, Edneia Amancio de Souza Ramos, Giseli Klassen, José Ederaldo Queiroz Telles, Cristhian Santos Oliveira, Maria Élida Alves Stefanello, Arthur J. Verhoeven, Ronald P. J. Oude Elferink, Alexandra Acco

**Affiliations:** 1 Department of Pharmacology, Biological Sciences Sector, Federal University of Parana – UFPR, PO Box 19031, Curitiba, PR 81531-980 Brazil; 2 Faculdades Pequeno Príncipe, Instituto de Pesquisa Pelé Pequeno Príncipe, Curitiba, PR Brazil; 3 Postgraduate Program in Pharmaceutical Sciences, University Vale of Itajaí, Itajaí, SC Brazil; 4 Pathology Department, Federal University of Parana, Curitiba, PR Brazil; 5 Medical Pathology Department, Federal University of Parana, Curitiba, PR Brazil; 6 Chemistry Department, Federal University of Parana, Curitiba, PR Brazil; 7 Academic Medical Center, Tytgat Institute for Liver and Intestinal Research, Amsterdam, The Netherlands

**Keywords:** Pharmacology, Cancer therapy, Oncology

## Abstract

Natural products have been recognized as important bioactive compounds on the basis of their wide biological properties. Here we investigated the antitumor effect and molecular mechanisms of the diterpene Fruticuline A (fruti) from *Salvia lachnostachys*, in human cancer cell lineages and Solid Ehrlich Carcinoma in mice. Fruti reduced MCF-7 and HepG2 proliferation by the reduction of Cyclin D1 levels and decreased NF-κB gene levels in both cell types. Furthermore, fruti also induced apoptosis in HepG2 cells, reduced Bcl-2 gene expression and induced necroptosis by increasing Ripk in MCF-7 cells. In mice, fruti prevented tumor development and reduced *Cyclin D1, Bcl-2* and *Rela* gene levels, and reduced the p-NF-κB/NF-κB ratio in tumor tissue. Furthermore, fruti induced necrosis and apoptosis, increased *N*-acetyl-β-D-glucosaminidase and TNF-α levels and reduced IL-10 and *Vegf* levels in tumor tissue. Collectively, fruti exerts antitumor effects through the inhibition of the NF-κB pathway, reducing Cyclin D1 and Bcl-2 levels. In vitro the apoptosis and necroptosis pathways are involved in the cellular death, whereas in vivo*,* cells undergo necrosis by increased tumor inflammation and reduction of angiogenesis. Thus, fruticuline A acts in tumor cells by multiple mechanisms and represents a promising molecule for drug development in cancer treatment.

Cancer remains the second leading cause of mortality throughout the world. The GLOBOCAN 2018 project estimated 9.3 million yearly deaths between 2012 and 2018 and 18.1 million of new cases in 2018 worldwide^1^. Cancer represents an important problem of public health, as the economic impact is significant and still increasing. In spite of the discovery of many antineoplastic drugs in the last decades and improved knowledge of cancer development, therapy is still hampered by the low efficacy of chemotherapeutics and by chemoresistance^2^.

Efforts have been made to better understand the biological basis of cancer progression and the specific pathways that are crucial in carcinogenesis. NF-κB represents one of the most important signaling pathways due to its transcription induction of many key-genes in cancer development, such as genes for proliferation (e.g. Cyclin D1 [CCND1], Cyclin E), apoptosis (e.g. Blc-2, Bcl-xL), angiogenesis (e.g. VEGF, IL-8, HIF1α), cell adhesion and metastasis (e.g. ICAM, MMP, iNOS), survival (e.g. Survivin) and inflammation (e.g. TNF-α, COX2, iNOS)^3^. Given studies showing the tumor-suppressive activity by inhibition of NF-κB^4,5^, this nuclear transcription factor became an attractive therapeutic strategy for novel antineoplastic compounds as several studies have revealed an important contribution of NF-κB to cancer progression and survival^6^. Therefore, the investigations of new candidates for cancer treatment are focusing in identifying molecules that target specific signaling pathways in tumor cells, providing a better therapeutic option for cancer.

Chemotherapeutic-induced side effects limit the treatment and often reduce quality of life for patients^7^. For this purpose, research has been focusing on finding molecules that target tumor cells and not normal cells. In this line, natural compounds, mostly from plants, have been used in traditional medicine for many diseases for thousands of years. These compounds represent a good alternative due to their wide biological properties and sometimes high affinity to biological receptors^8^. Almost 80% of all drugs approved for cancer therapy by the US Food and Drug Administration during the last three decades are natural products per se or semi-synthetic products^9^.

A variety of products with anticancer potential can be found in nature, and among them is fruticuline A (fruti). Fruti is a quinone diterpene and one of the major compounds in the ethanolic extract from leaves of *Salvia lachnostachys* (EES), recently characterized (Fig. 1)^10^. Previously, our group demonstrated the antitumor effect of EES in a solid tumor animal model, the Ehrlich tumor in mice^11^. Furthermore, several biological properties of fruti were already described, such as antinociceptive, anti-inflammatory and antidepressive effects in vivo and cytotoxicity against a range of cancer cell lineages in vitro^10,12,13^. However, there are no data on the in vivo antitumor effect of purified fruti. This study investigated this aspect of fruti as well as its action mechanism, using in vitro neoplastic cell lineages and in vivo Solid Ehrlich Carcinoma (SEC) of mice.

**Figure 1:**
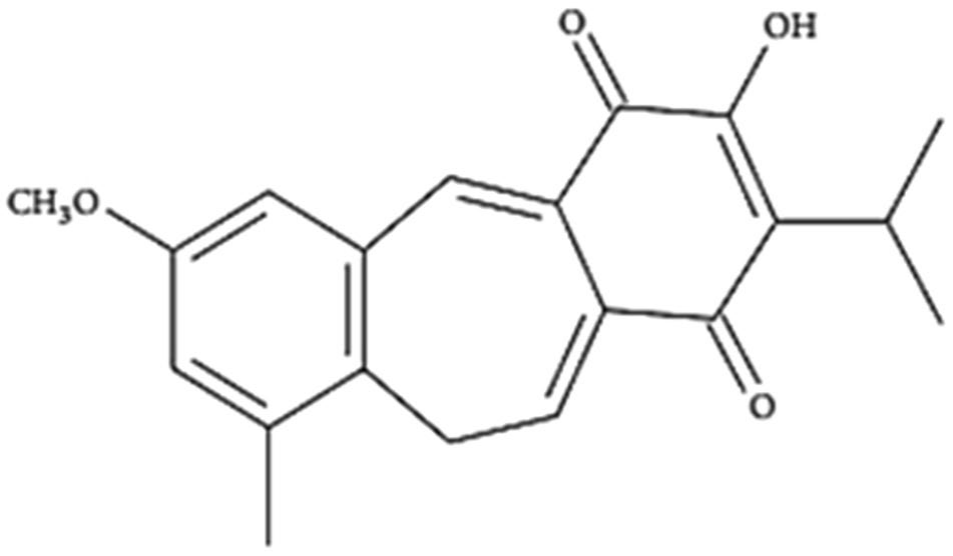
Chemical structure of fruticuline A^15^.

## Results

### Isolation and identification of fruti

Pure fruticuline A was obtained by preparative HPLC and analyzed by NMR (^1^H, HSQC, HMBC). The NMR data were identical with those previously reported^14, 15^. The ^1^H NMR (Supplementary Fig. S1) showed the typical signals of fruticuline A: two doublets at δ_H_ 1.26 (six hydrogens) and δ_H_ 3.14 (two hydrogens); two singlets at δ_H_ 2.44 and 3.79 (three hydrogens each one); an septet at δ_H_ 3.37 (one hydrogen); a multiplet at δ_H_ 6.86–6.98 (three hydrogens); two singlets at δ_H_ 7.66 (hydroxyl group) and 8.16 (one hydrogen). Besides the solvent signal (δ_H_ 7.27), no other signals were observed, confirming the absence of impurities, as aliphatic compounds or other aromatic diterpene, such as fruticuline B. The yield of fruticuline A from EES was around 3%.

### Fruti has cytotoxicity on human cancer cells but does not induce oxidative stress in MCF-7 cells

**Figure 2:**
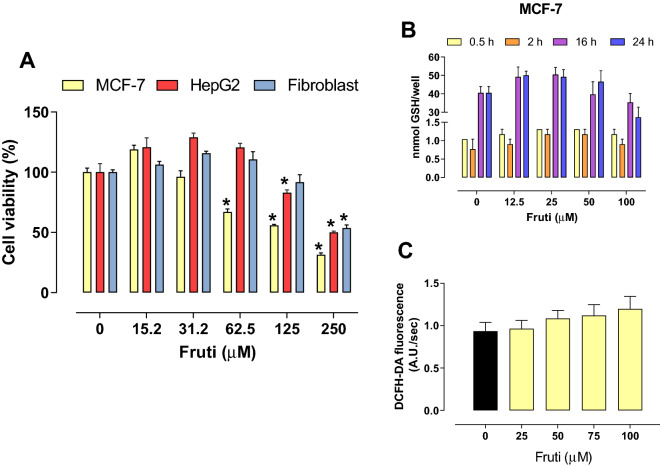
Cell viability (**A**), GSH levels (**B**) and ROS production (**C**). Panel (**A**)**:** MCF-7, HepG2 and Fibroblasts cells were incubated with vehicle (0) or fruti at 15.2, 31.2, 62.5, 125 and 250 µM for 24 h. Panel (**B**): For GSH measurement MCF-7 cells were incubated with vehicle (0) or fruti at 12.5, 25, 50 and 100 µM for 0.5, 2, 16 and 24 h. Panel (**C**): ROS levels were monitored in MCF-7 cells loaded with DCFH-DA that were incubated with vehicle (0) or fruti (25, 50, 75 and 100 µM) for 60 min. Results are expressed as mean ± S.E.M. (n = 3) and were analyzed by one-way ANOVA followed by Newman Keuls post hoc test. *p < 0.05 when compared to vehicle group.

Fruti at the concentration of 62.5, 125 and 250 µM inhibited MCF-7 viability in 33, 44 and 69% respectively, when compared to the vehicle group. In HepG2 cells the inhibition occurred with Fruti at 125 and 250 µM (17 and 50%, respectively), whereas in fibroblasts, only the concentration of 250 µM reduced viability by 46%, compared to vehicle group (Fig. 2A). The IC_50_ of Fruti was 57.7, 119.4 and 145.3 µM for MCF-7, HepG2 and fibroblasts, respectively. Nevertheless, no changes were observed in GSH levels after 0.5, 2, 16 and 24 h of incubation with Fruti (Fig. 2B) and in ROS levels in MCF-7 cells compared to the vehicle group (Fig. 2C).

### Fruti decreases *CYCLIN D1* and *BCL-2* but not VEGFA proteins in human cancer cells

**Figure 3:**
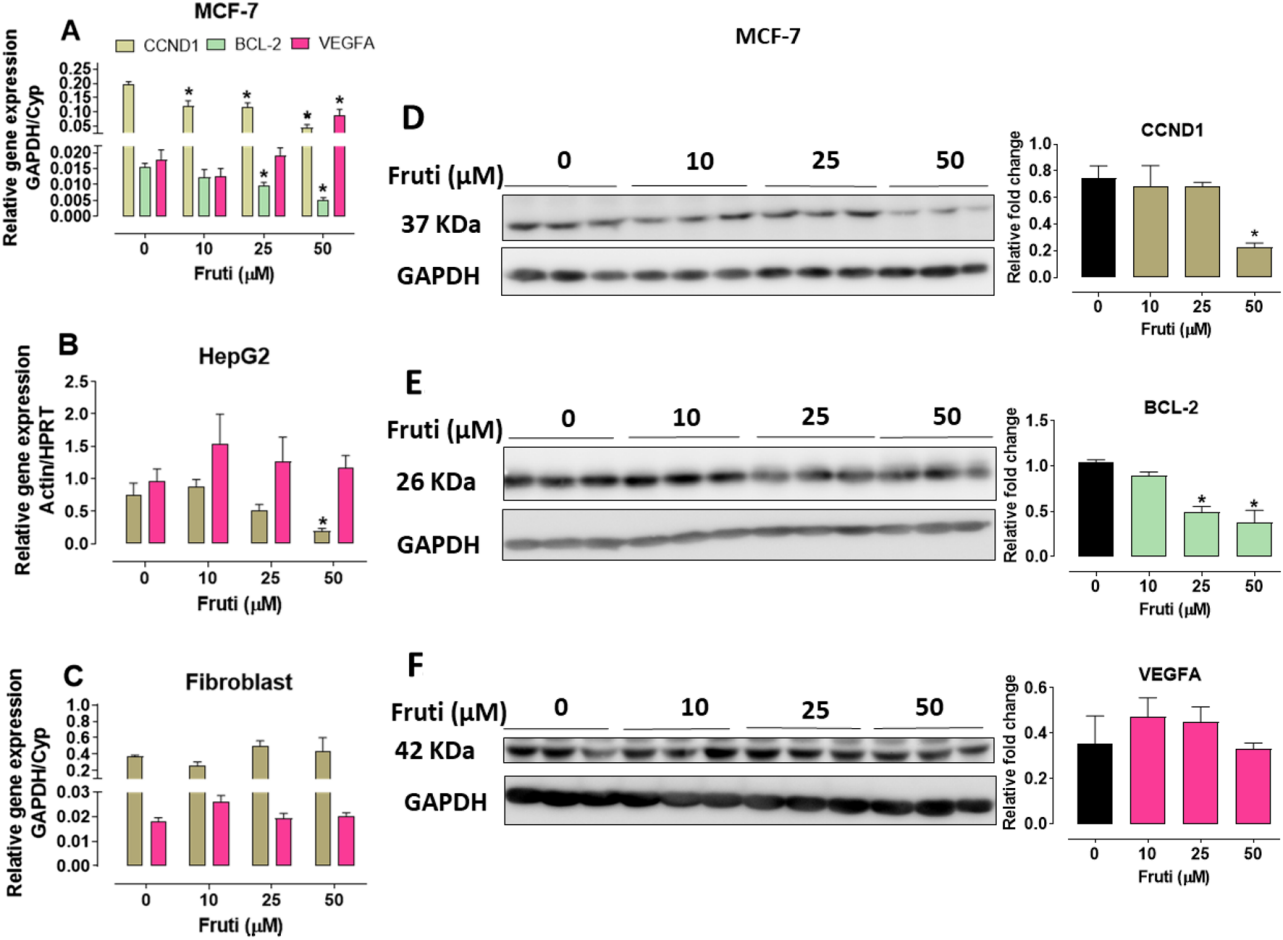
Gene expression of *CCND1*, *BCL-2* and *VEGFA* in MCF-7 (**A**), HepG2 (**B**) and fibroblasts (**C**) and proteins levels in MCF-7 (D, E, F). Panel (**A**), (**B**) and (**C**): *CCND1, BLC-2* and *VEGFA* expression in MCF-7, HepG2 and Fibroblasts cells, respectively; Panel (**D**)**,** (**E**) and (**F**): CCND1, BCL-2 and VEGFA proteins levels in MCF-7 cells by Western blot assay. The blot images have been cropped for conciseness. The full-size blots are presented in Supplementary Fig. S2. Cells were incubated with vehicle (0) or fruti 10, 25 and 50 µM for 24 h. Results are expressed as mean ± S.E.M. (n = 4–6) and were analyzed by one-way ANOVA followed by Newman Keuls post hoc test. *p < 0.05 when compared to vehicle group.

The expression of *CYCLIN D1 (CCND1* gene*)* decreased in human cancer cells (MCF-7 and HepG2) after 24 h in the presence of 50 µM fruti when compared to vehicle group (MCF-7: 76%; HepG2: 74%) (Fig. 3A,B). In addition, fruti at 25 and 50 µM reduced *BCL-2* expression in MCF-7 cells by 36 and 66%, respectively (Fig. 3A). However, at 50 µM there was an increase in *VEGFA* gene in MCF-7 cells (Fig. 3A). No differences were found in fibroblasts (Fig. 3C). Furthermore, the expression of TNF-α was not detected in these cell lines.

Western blot analysis demonstrated a reduction on CYCLIN (CCND1) protein in MCF-7 cells treated with 50 µM fruti (Fig. 3D). Furthermore, fruti at 25 and 50 µM decreased BCL-2 levels (Fig. 3E), and no difference was observed in VEGFA levels (Fig. 3F).

### Fruti treatment induces apoptosis and necroptosis in tumor cells

**Figure 4:**
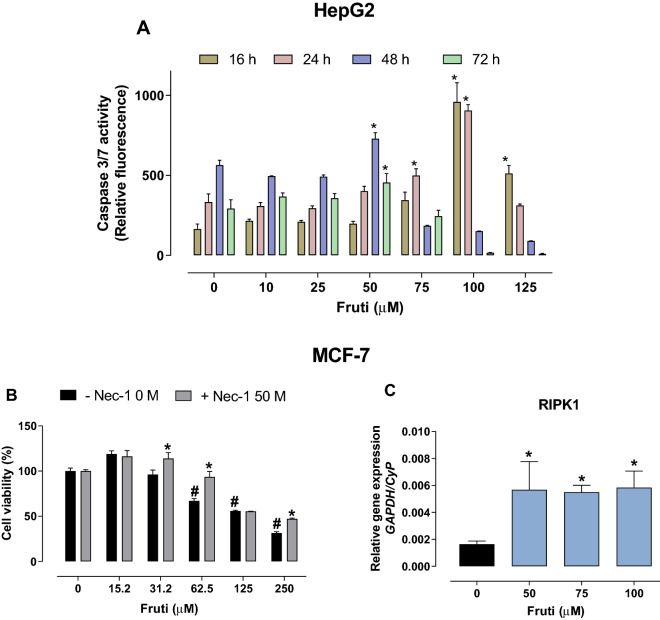
Caspase 3/7 activity in HepG2 cells (**A**), cell viability in the presence of Necrostatin-1 (**B**) and *RIPK1* gene expression (**C**) in MCF-7 cells. For caspase 3/7 activity HepG2 cells were incubated with vehicle (0) or fruti at 10, 25, 50, 75, 100 and 125 µM for 16, 24, 48 and 72 h (Panel **A**). For cell viability MCF-7 cells were incubated with vehicle (0) or fruti at 15.2, 31.2, 62.5, 125 and 250 µM for 24 h with or without Necrostatin-1 (Nec-1) (Panel **B**). For measurement of *RIPK1* gene expression MCF-7 cells were incubated with vehicle (0) or fruti at 50, 75 and 100 µM for 24 h (Panel **C**). Results are expressed as mean ± S.E.M. (n = 3) and were analyzed by one-away ANOVA followed by Newman Keuls post hoc test. * *p* < 0.05 when compared to vehicle group (Panel **A** and **C**). # and *p < 0.05 when compared to vehicle group (0 µM) in absence of Necrostatin-1 (- Nec-1) and fruti concentrations in the absence of Nec-1, respectively (Panel **B**).

We observed a time- and dose-dependent increase in Caspase 3/7 activity upon incubation with fruti in HepG2 cells. At 16 h of incubation with 100 µM and 125 µM fruti augmented caspase 3/7 activity by 489 and 213%, respectively. At 24 h of incubation with 75 µM and 100 µM fruti increased caspase 3/7 50 and 172%, respectively. At 48 and 72 h of incubation, 50 µM fruti increased activity by 29 and 55%, respectively. Finally, at 48 and 72 h a substantial fraction of the cells incubated with fruti in concentrations higher than 100 µM were dead, and therefore caspase 3/7 activity was not detectable anymore (Fig. 4A).

To evaluate if necroptosis is involved in the mechanism of fruit-induced cell death, MCF-7 cells were incubated with Necrostatin-1 (Nec-1), an inhibitor of RIPK1. Nec-1 partially reversed the inhibition of the MCF-7 cells proliferation in the presence of fruti (at 62.5 and 250 µM after 24 h of incubation) (Fig. 4B). The same was observed in HepG2 cells (Supplementary Fig. S3). In addition, fruti at 50, 75 and 100 µM increased *RIPK1* expression in MCF-7 cells compared to the vehicle group (0) (with 255, 236 and 265%, respectively) (Fig. 4C).

### Fruti inhibits NF-κB1 gene expression but not pJNK and STAT3 proteins in MCF-7 cells

**Figure 5:**
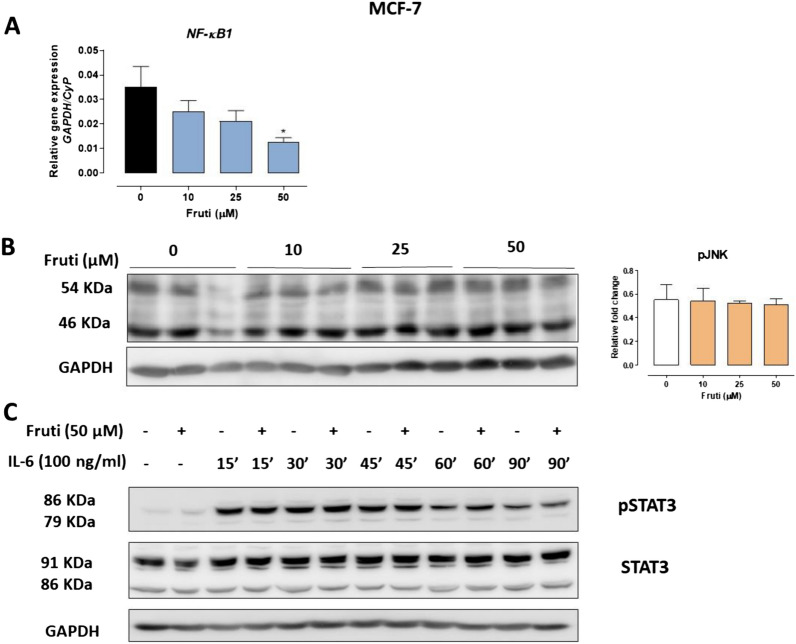
*NF-kB1* gene expression (Panel **A**) and protein levels of pJNK (Panel **B**), pSTAT3 and STAT3 (Panel **C**) in MCF-7 cells. For pJNK and *NF-κB1* analysis, cells were incubated with vehicle (0) or fruti at 10, 25 and 50 µM for 24 h. For pSTAT3 and STAT3 analysis, cells were incubated with vehicle (0) or fruti at 50 µM in the presence or absence of IL-6 (100 ng ml^−1^) for 15 until 90 min. The blot images have been cropped for conciseness. The full-size blots are presented in Supplementary Fig. S6. Results are expressed as mean ± S.E.M. (n = 3) and were analyzed by one-way ANOVA followed by Newman Keuls post hoc test. * *p* < 0.05 when compared to vehicle group.

Fruti at 50 µM decreased NF-κB1 gene expression (63%) in MCF-7 cells after 24 h of incubation compared to the control group (Fig. 5A). Furthermore, in HepG2 cells fruti at 25 and 50 µM also inhibited NF-κB1 expression by 73 and 65%, respectively (Supplementary Fig. S4). Nevertheless, no differences were observed on pJNK, pSTAT3 and STAT3 levels in MCF-7 cells incubated with fruti (Fig. 5B,C), and this also held for STAT3 gene expression in MCF-7 and HepG2 cells (Supplementary Fig. S5).

### Treatment with fruti inhibits Ehrlich tumor development, induces inflammation, apoptosis and necrosis in tumor tissue

**Figure 6:**
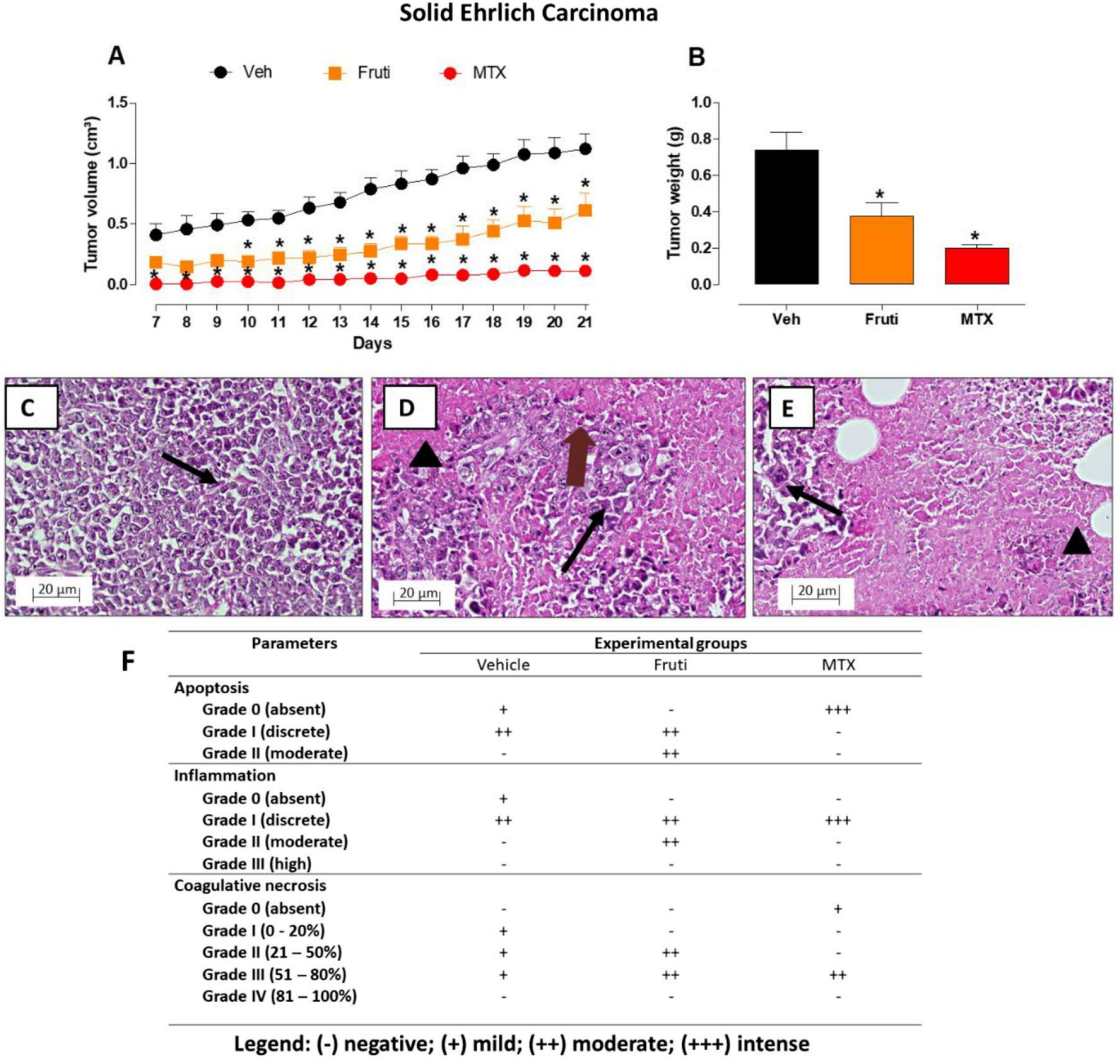
Solid Ehrlich tumor development (**A**), tumor weight (**B**) and tumor histology (**C–F**) and quantification (**F**) in mice. Animals were treated with vehicle p.o (Panel **C**), fruti 3 mg kg^−1^, p.o. (Panel **D**), or methotrexate, MTX, 2.5 mg/kg, i.p. (Panel **E**) for 21 days. Results are expressed as mean ± S.E.M. (n = 6–9) and were analyzed by two-way ANOVA followed by Bonferroni post hoc test (**A**) or one-way ANOVA followed by Newman Keuls post-hoc test (**B**). *p < 0.05 when compared to vehicle group. Black Arrows and head arrows indicated Ehrlich viable cells and coagulative necrosis area, respectively. Brown arrow indicates apoptotic cells. Samples were observed in optical microscope at 20× (scale bar 20 µm).

In order to test the effect of purified fruti on tumor development in vivo, mice were inoculated with Ehrlich ascites cells and either treated with fruti or vehicle. Treatment with fruti (3 mg kg^−1^) significantly inhibited tumor growth from day 10 until the end of experiment, when compared to the vehicle group (Fig. 6A). MTX, used as positive control, also inhibited tumor growth until day 21. In addition, the tumor weight on day 21 was significantly reduced (49%) by fruti treatment (Fig. 6B).

Tumor tissue of animals treated with vehicle displayed viable Ehrlich cells, small areas of coagulative necrosis and discrete inflammation observed by polymorfonuclear cells infiltration and discrete apoptosis (Fig. 6C). Tumor tissue of animals treated with fruti had more coagulative necrosis area, less viable Ehrlich cells, higher inflammation and moderate apoptosis when compared to the vehicle group (Fig. 6D). Furthermore, MTX treatment also induced discrete inflammation and coagulative necrosis, but no apoptosis in tumor tissue (Fig. 6E). The tumor histology quantification is summarized in Fig. 6F.

### Fruti decreases *Ccnd1, Bcl-2, Vegfa* and *Rela* gene expression in tumor tissue

**Figure 7:**
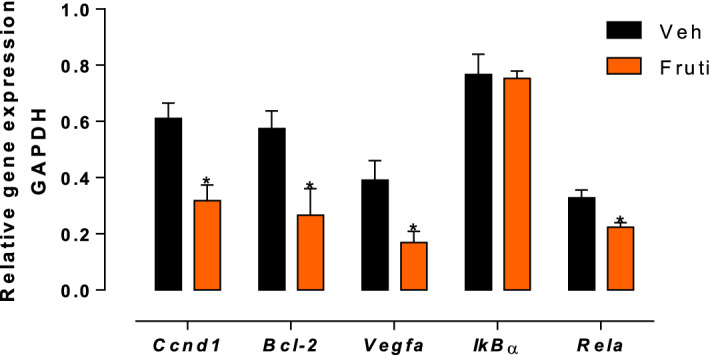
Gene expression of *Ccnd1*, *Bcl-2*, *Vegfa*, *Ikbα* and *Rela* in tumor tissue. Animals were treated with fruti (3 mg kg^−1^, p.o.) or vehicle (Veh, 0.1% tween 20 in distilled water, 10 mL kg^−1^, p.o.) for 21 days. Results are expressed as mean ± S.E.M. (n = 4–5) and were analyzed by Student t-test. * *p* < 0.05 when compared to vehicle group.

Fruti reduced *Ccnd1 (Cyclin D1)*, *Bcl-2*, *Vegfa* and *Rela* gene expression by 48, 54, 58 and 32%, respectively, when compared to vehicle group, whereas no difference was found on *IkBα* expression (Fig. 7).

### Fruti reduces NF-κB levels in tumor tissue

**Figure 8:**
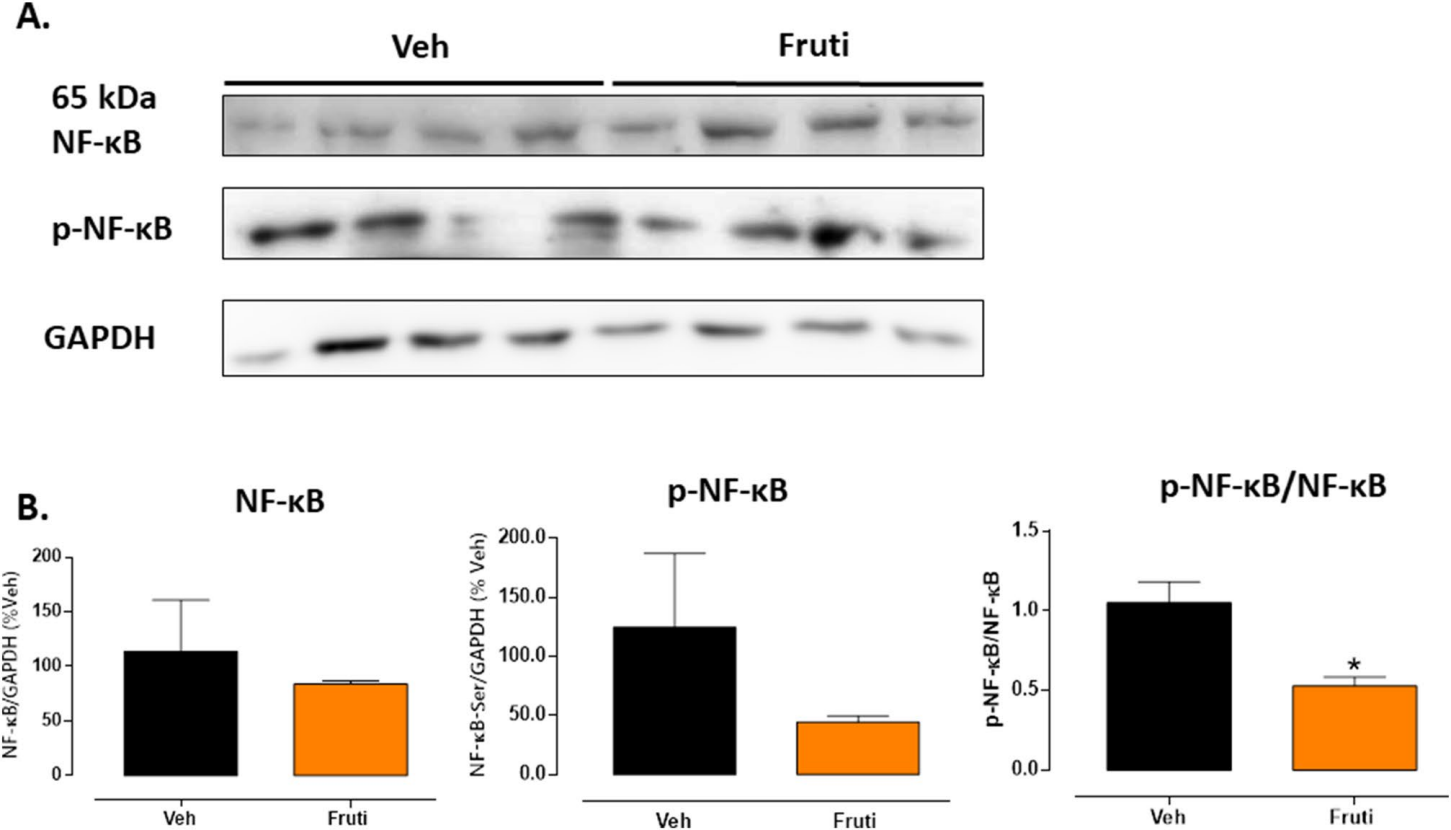
Protein levels of NF-κB and p-NF-κB in tumor tissue (Panel **A**). Animals were treated with fruti (3 mg kg^−1^, p.o.) or vehicle (Veh, 0.1% tween 20 in distilled water, 10 mL kg^−1^, p.o.) for 21 days. The blot images have been cropped for conciseness. The full-size blots are presented in Supplementary Fig. S8. Panel (**B**) represents protein levels quantification of NF-κB/GAPDH, p-NF-κB/GAPDH and the ratio p-NF-κB/NF-κB, respectively. Results are expressed as mean ± S.E.M. (n = 4) and were analyzed by one-way ANOVA followed by by Student t-test. * *p* < 0.05 when compared to vehicle group.

After 21 days of treatment, despite the total protein level of phosphorylated NF-κB and total NF-κB did not differ among groups, fruti reduced in 49% the p-NF-κB/NF-κB ratio in tumor tissue (Fig. 8).

### Fruti induces oxidative stress and inflammation in tumor tissue

Fruti treatment increased ROS, *N*-acetyl-β-D-glucosaminidase (NAG) and TNF-α levels in tumor tissue by 15, 29 and 79%, respectively, and reduced IL-10 by 68%, compared to vehicle group. No differences were observed in the other inflammatory parameters (Table 1).

**Table 1:** Oxidative stress and inflammatory parameters in Ehrlich tumor tissue.

Parameters	Experimental groups	
	Veh	Fruti
GSH (µg g of tissue^−1^)	197.51 ± 14.44	239.01 ± 39.71
DCFH-DA fluorescence	84.06 ± 1.02	96.69 ± 3.19*
MPO (µmol min^−1^ g of tissue^−1^)	164.07 ± 15.88	145.05 ± 11.16
NAG (µmol min^−1^ g of tissue^−1^)	20.03 ± 1.30	25.89 ± 2.89*
NO (µmol g of tissue^−1^)	8.13 ± 1.18	6.50 ± 0.58
TNF-α (pg mL^−1^)	1525.00 ± 212.05	2735.00 ± 352.09*
IL-4 (pg mL^−1^)	458.07 ± 88.78	456.09 ± 109.01
IL-6 (pg mL^−1^)	65.55 ± 5.13	96.63 ± 23.16
IL-10 (pg ml^−1^)	3232.00 ± 521.00	1050.00 ± 255.05*

Animals were treated with fruti (3 mg kg^−1^, p.o.) or vehicle (Veh, 0.1% tween 20 in distilled water, 10 mL kg^−1^, p.o.) for 21 days. Results are expressed as mean ± S.E.M. (n = 5–7) and were analyzed by Student t-test.

* *p* < 0.05 when compared to vehicle group.

### Fruti does not induce hematological toxicity and increases monocytes

As depicted on Supplementary Table S1, fruti treatment induced a slight decrease in ALT and increased in monocyte numbers, compared to the vehicle group. MTX treatment reduced total protein, globulin, lymphocytes and hemoglobin levels and increased monocytes compared to vehicle and naïve group. In addition, no toxicity was observed in liver tissue upon Fruti treatment (Supplementary Fig. S8).

## Discussion

**Figure 9:**
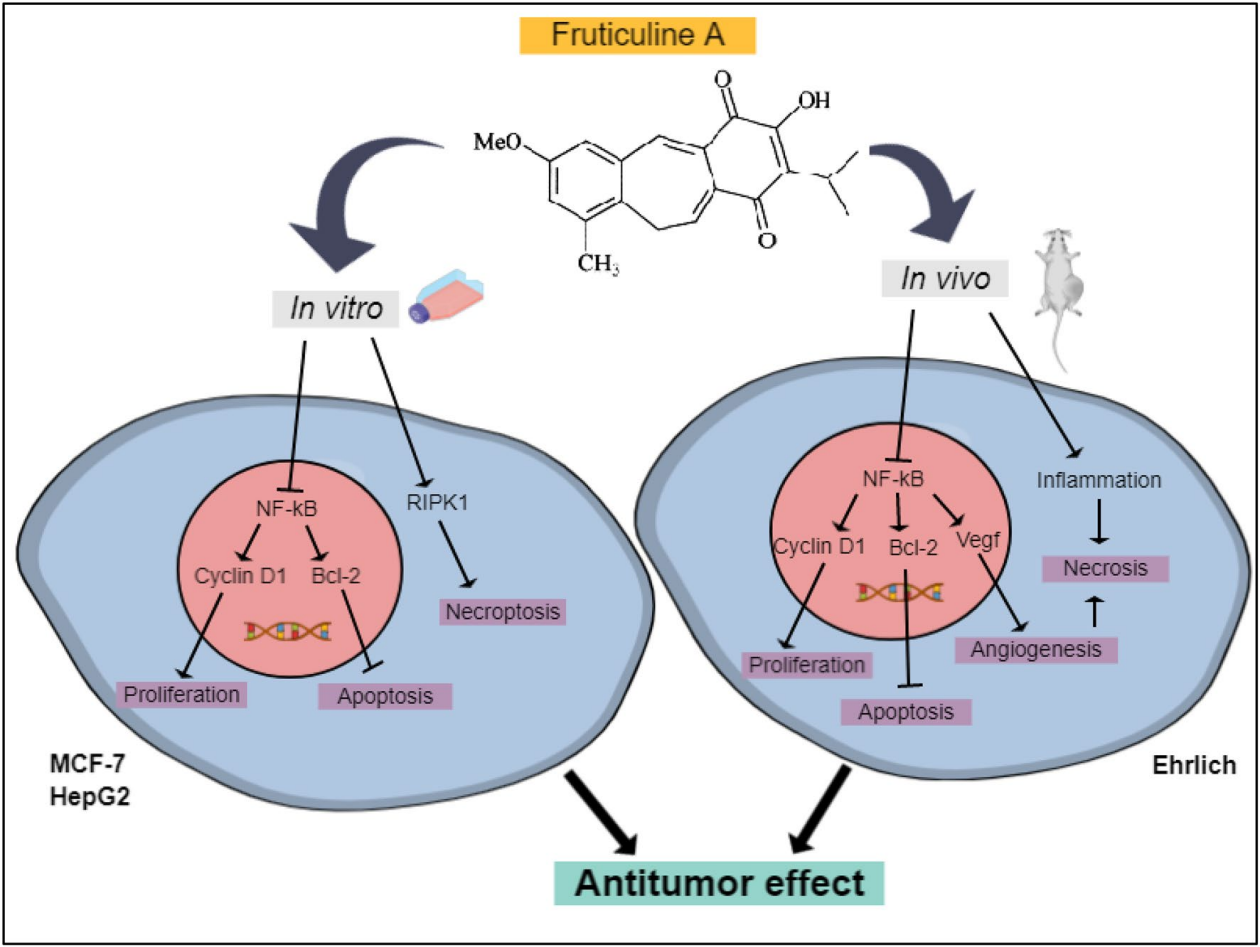
Schematic summary of the results.

Fruticuline A is one of the major constituents of the ethanolic extract of *Salvia lachnostachys* (EES) leaves, which was demonstrated to have antitumor effect against Solid Ehrlich Carcinoma (SEC) in mice^11^. Fruti is a diterpene quinone and has some biological properties already described, such as antinociceptive, anti-inflammatory, antidepressive and antimicrobial^12,13,16^. Cytotoxicity of fruti against a range of human cancer cell lines has also been demonstrated, including U251 (glioma, central nervous system), UACC-62 (melanoma), MCF-7 (breast), NCI-ADR/RES (ovarian-resistant), NCI-H460 (lung, non-small-cell), PC-3 (prostate), OVCAR-3 (ovarian) and HT-29 (colon)^10^. However, none of these studies investigated the antitumor effect of fruti and its action mechanism. Thus, the present study was the first one to investigate the antitumor effect of pure fruti in vivo and to elucidate the mechanism of action in different tumor cell lineages summarized in Fig. 9*.*

Fruti was capable of inhibiting proliferation of tumor cells (MCF-7 and HepG2) indicating its antiproliferative effect. As fibroblasts are non-tumor cells that surround tumor tissue, we then investigate whether fruti could inhibit the proliferation of this cell type. A reduction of proliferation was only observed at high concentration of fruti (250 µM), but no alterations in expression of genes related to proliferation, apoptosis and angiogenesis in these cells. Thus, we hypothesized that instead of damage to normal cells, fruti not only inhibits the proliferation but also induces cell death only in tumor cells. For this reason, we focused to investigate its antitumor effect and its mechanism in tumor cells.

Fruti inhibited *CCND1 (CYCLIN D1*) and *BCL-2* gene expression in MCF-7 cells. Cyclin D1 is a protein that regulates proliferation and is found in cytoplasm and nuclei^17^, whereas Bcl-2 is a regulatory anti-apoptotic proteinthat determines the response of cancer cells to chemotherapeutics^18^. Both genes can be transcriptionally regulated by STAT3 and/or NF-κB, which play an important role in the development of carcinogenesis^3,19,20^. Despite the fact that fruti did not change STAT3 levels, it decreased NF-κB1 gene expression. NF-κB comprises a family of transcription factors, including RELA, RELB, NF-κB1, NF-κB2, and IκBα, which are inducible by a variety of stimuli. Protein products of these genes lead to activation of p65, p50 and p52. The nuclear translocation of NF-κB complexes p65/p50 and p52/RELB is triggered by degradation of IκBs, particularly IκBα, leading to the nuclear translocation of several NF-κB complexes, predominantly the p50/RELA dimer^21^. Consequently, these complexes regulate the transcription of genes in the nuclei thereby mediating several cellular functions such as proliferation (e.g. Cyclin D1, Cyclin E, IL-6), tumor promotion (e.g. VCAM, ICAM, iNOS, COX 2), apoptosis (e.g. Bcl-2, Bcl-xL, Survivin) and inflammation (e.g. TNF-α, COX2, iNOS)^3,22^. Thus, our results suggest the involvement of the NF-κB pathway on the regulation of Cyclin D1 and Bcl-2 gene expression induced by fruti. However, a global inhibition of NF-κB is not possible, as it would induce genetic instability^6^. In fact, we observed a partial decrease in NF-κB, in the order of 63 and 65% in MCF-7 and HepG2 cells, respectively, compared to the control group. In addition, it was already described that some diterpenes such as excisanin A and kamebakaurin from *Isodon japonicus*, inhibit NF-κB in inflammatory processes^23,24^. Therefore, it is possible that the antitumor effect of fruti is dependent of NF-κB inhibition.

The mechanism of cellular death in neoplastic cells can be the result of programmed cellular death (apoptosis), necrosis or necroptosis. These pathways are induced by different signals, such as DNA damage, oxidative stress, hypoxia and/or ligands of death receptor family (i.e. TNF-α)^25^. As fruti is a quinone compound, which are known inducers of GSH adduct formation and oxidative stress^16,26^, we measured GSH content and ROS production on MCF-7 cells. However, curiously, neither short nor longer incubation times depleted GSH, showing no involvement of cellular death by oxidative stress. On the other hand, the c-Jun N-terminal kinase (JNK) pathway is also involved in the regulation of cell death. JNK forms an important subgroup of the mitogen-activated protein kinases (MAPK) superfamily and are mainly activated by oxidative stress and/or TNF-α, being a key promoter for cellular suicide by apoptosis or necrosis^27^. The programmed cell death, on the other hand, has a variety of pathways and proteins that regulate or trigger apoptosis, such as the anti- (e.g. Bcl-2, Bcl-xL) or pro- (e.g. Bax, Bid) apoptotic proteins and caspases effectors (e.g. caspase 3 and 7)^28^. While TNF-α was not detected in the cells and no difference was observed in pJNK levels, apoptosis induced by fruti, was probably due to the observed reduction on Bcl-2 levels. In addition to apoptosis, we also observed that fruti enhanced RIPK1 gene expression at higher concentrations. RIPK1 is the target for necroptosis pathway, a form of regulated necrotic cell death. Nec-1 has been used as a tool to investigate necroptosis pathway due to its ability in inhibit RIPK1 activity^29^. In fact, the antiproliferative effect of fruti in MCF-7 cells was reversed in the presence of Nec-1, confirming the involvement of RIPK1 in the cytotoxicity of fruti. Thus, in vitro fruti also induced cellular death through the necroptosis pathway.

In vivo, the antitumor effect of fruti was confirmed by the effective prevention of tumor development in animals with SEC. In tumor tissue of animals treated with fruti we observed a reduction of *Cyclin D1*, *Bcl-2* and *Rela* (a protein from NF-κB pathway) expression and of p-NF-κB/NF-κB protein ratio, in line with our observations from in vitro experiments. However, with respect to VEGF gene expression the results were different. Reduction on *Vegf* expression was observed with fruti treatment in vivo, whereas in vitro only the MCF-7 exhibited an increase in *VEGF* expression with higher concentrations of fruti, but without concomitant increase in VEGF protein. This difference may due to the fact that VEGFA mRNA is highly labile under normal oxygen and nutrient conditions with a half-life of 15–40 min in vitro^30^. The reduction of *Vegf* expression in vivo can reflect a reduction of angiogenesis and nutrition to tumor tissue, which induces cellular death by starvation resulting in coagulative necrosis^31^. Therefore, beyond the tumor cell death through apoptosis induced by fruti in SEC, it also has anti-angiogenic properties in solid tumors inducing to cell death by coagulative necrosis.

As inflammation is intrinsically correlated with cancer development^32^, we investigated some cytokines in tumor tissue. Interestingly, there was an increase in tumor TNF-α and decrease in IL-10 levels in animals treated with fruti, showing a pro-inflammatory effect of fruti. The increase in TNF-α levels can be correlated with an increase in blood monocytes, which could be a source for TNF-α production^33^. In addition, *Rela* expression in tumor tissue was partially inhibited by fruti, despite no changes were observed in *IkBα* expression, suggesting that the inflammation induced by fruti is not dependent of NF-κB pathway. This indicates a compensatory mechanism of fruti treatment in the steady state of IkBα/NF-κB activation, disturbing only the expression of the active form *Rela*^34^. In addition to the enhanced inflammatory environment in tumor tissue, we observed a slight increase in ROS levels, which can also be triggered by inflammatory mediators. Indeed, the tumor histology showed more inflammatory cells, coagulative necrotic cells and apoptosis, and less Ehrlich viable cells, in tumor of fruti-treated mice compared to the control group. Thus, the increase in tumor inflammation can also induce tumor cell death or can be a result of the necrosis^35,36^ induced by the fruti**.**

The inflammatory tumor microenvironment can be the explanation for some different results observed in in vitro and in vivo experiments with fruti, for instance the VEGF. Due to the pro-inflammatory effect of fruti we hypothesized that in vivo the tumor microenvironment changes the response to antitumor agents, compared to in vitro observations. The long-lasting treatment in vivo can also change the mechanism of cellular death. Furthermore, the difference in tumor cell lineages studied in vitro (MCF-7 and HepG2) and in vivo (Ehrlich) should be taken into account in the interpretation of the results.

Despite the cytotoxic effects of fruti in tumor cells, it did not induce systemic toxicity in mice, observed by the hematological and biochemical parameters. Moreover, no difference among treatments was observed in body and organ weight (data not shown) and in liver histology. Previously, our group showed that the source of fruti, the *S. lachnostachys* ethanolic extract, had no toxicity, with a LD_50_ higher than 2000 mg kg^−1^^11^. On the other hand, MTX, the chemotherapy compound used as positive control, has oral LD_50_ in mice of 146 mg kg^−1^^37^. MTX treatment in mice bearing Ehrlich tumor produced systemic and hematologic adverse effects. As fruti also had antitumor effect, this can make fruti, or an improved derivative of fruti, a good candidate to treat solid cancers with less toxic effects.

In conclusion, fruticuline A exerts antitumor effects through inhibition of the NF-κB pathway, reducing Cyclin D1 and Bcl-2 levels. The effects depend on both the used cell lineages and the experimental conditions. In MCF-7 and HepG2 cells the apoptosis and necroptosis pathways are involved in cell death, whereas in the Ehrlich tumor in mice, due to the long-term treatment and tumor microenvironment, cells undergo necrosis by the increase in tumor inflammation and reduction of angiogenesis. Thus, fruticuline A acts on tumor cells by a combination of two mechanisms and represents a promising molecule for being used directly or in a modified formulation in cancer therapy, especially against solid mammary and hepatic tumors.

## Material and methods

### Isolation of fruticuline A by HPLC and NMR

A detailed procedure of isolation and identification of fruticuline A from *Salvia lachnostachys* (SisGen AA49C69) was previously reported^10^. Briefly, dried and powdered leaves of *S. lachnostachys* (415.3 g) were extracted with hexane and ethanol, at room temperature. The ethanol extract (EES, 45.0 g) was submitted to silica gel vacuum liquid chromatography and eluted with hexane, hexane:dichloromethane 1:1, pure dichloromethane, acetone, and methanol. The fraction eluted with hexane (1.8 g) was subjected to silica gel column chromatography, eluted with mixtures of hexane:dichloromethane in increasing polarity, to give 14 subfractions.

Subfraction 6 revealed to contain mainly impure fruticuline A (458.4 mg). An aliquot (80 mg) was purified by HLPC (Rt = 8.21 min, methanol:acetonitrile 50:50, isocratic, semipreparative Nucleosil 100–5 C18 column, 250 × 10 mm), yelding pure fruticuline A (58.8 mg). Fruticuline A was identified by 1D and 2D Nuclear Magnetic Resonance [Bruker Avance 400 spectrometer, using deutered chloroform (CDCl_3_) as solvent], and compared with previously reported data^14,15^.

### In vitro experiments

#### Culture cells

Human MCF-7, HepG2 and Fibroblasts cells were cultured in Dulbecco’s Modified Eagle’s Medium (DMEM), supplemented with 10% fetal bovine serum, 4 mmol L^−1^ of *L*-glutamine and a mixture of antibiotics (5 mg mL^−1^ of streptomycin and 5 mg mL^−1^ penicillin). Cells were incubated at 37 °C in humidified atmosphere containing 5% CO_2_. After cells reached ~ 80% of confluence, cells were harvested and seeded in a 96 well plate or 12 well plate, according to the experimental protocol.

#### WST-1 viability assay

The WST-1 viability assay is a colorimetric assay for the quantification of cell viability, based on the cleavage of the tetrazolium salt WST-1 by mitochondrial dehydrogenase in viable cells producing a colorimetric product, formazan (CELLPRO-Roche, Mannheim, Germany). Briefly, 5 × 10^3^ of MCF-7 cells/well were added in 96 wells plate. Cells were incubated with vehicle (0) or fruti (31.2, 62.5, 125 and 250 µM) dissolved in 0.1% DMSO/DMEM for 24 h. Then, the medium was replaced by new medium to which 10 µL of WST-1was added and the cells were incubated for 2 h at 37 °C in humidified atmosphere 5% CO_2_. Additionally, fibroblasts were also incubated with fruti to evaluate the cytotoxicity of fruti in non-transformed cells. Viability was measured by spectrophotometer at 450 and 690 nm, after 1 min shaking the plate to equilibrate the intracellular formazan. The results were expressed as cell viability (%). In addition, to evaluate the necroptosis pathway, the same procedure described above was performed, including the incubation of cells with 50 µM necrostatin-1 (Nec-1) (Sigma-Aldrich, St. Louis, Missouri, EUA), an inhibitor of receptor-associated kinase 1 (RIPK1).

#### Oxidative stress parameter assays

To perform reduced glutathione (GSH) measurement, MCF-7 cells were seeded in 96 wells plate until reaching 80% of confluence. Vehicle (0) or fruti (12.5, 25, 50 and 100 µM) was incubated for 0.5, 2, 16 and 24 h to better evaluate if alterations in quinone concentration over a period of time would deplete this peptide. After the incubation, cells were scratched in the presence of iced cold 10% perchloric acid and centrifuged for 10 min at 14,000×*g* 4 °C. To increase the pH to 8, 3.0 M K_2_PO_4_ was added in the supernatant and samples were immediately frozen in liquid nitrogen. After 30 min samples were thawn and incubated with work solution (DTNB and NADPH) diluted in GSH buffer (0.1 M Na_2_HPO_4_.2H_2_O, 0.1 M NaH_2_PO_4_.2H_2_O and 0.05 M Na_2_EDTA.2H_2_O) in a 96 wells plate. The reaction was started by addition of glutathione reductase (GR) and the absorbance was monitored at 412 nm at 37 °C on a Clariostar (BMG LabTech, Cary, USA) analyzer. The results were interpolated with GSH standards and were expressed in nmol GSH/well^38^.

Additionally, to measure total reactive oxygen species (ROS), MCF-7 cells were seeded in a black 96 wells plate. After 24 h cells were briefly washed with DMEM without phenol red and supplemented with 1.9 g L^−1^ NaHCO_3_, 5 mM glucose and 0.1% fetal bovine serum. 5 µM DCFH-DA was added to monitor ROS production following addition of fruti concentrations (25, 50, 75 and 100 µM), dissolved in 0.1% DMSO/DMEM (-) phenol red. The fluorescence was monitored on a Clariostar analyzer at 488/20, 520/20 nm at 37 °C, 5% CO_2_. Results were expressed as DCFH-DA fluorescence (AUC/sec)^39^.

#### Total RNA isolation and RT-qPCR

Human MCF-7, HepG2 and Fibroblast cells were seeded in 12 wells plate. After reaching confluence, cells were incubated with vehicle (0) or fruti at 10, 25, 50, 75 or 100 µM for 24 h (concentrations previously chosen by WST1 assay screening). Total RNA was extracted from cultured cells using Trizol reagent (Sigma-Aldrich, St. Louis, Missouri, USA) and measured by Nanodrop 100 (Thermo Scientific, Waltham, Massachusetts, USA). Complementary DNA was synthesized from 2 µg total RNA with an oligo-dT, random hexamers primers and deoxythymidine oligomer, Ribolock RNAse inhibitor and RevertAid reverse transcriptase. Real-time polymerase chain reaction was performed in a Lightcycler apparatus (Roche, Mannheim, Germany) using the sensiFAST SYBR No-ROX kit (Bioline, London, UK). Initial fluorescent values were calculated by LinRegPCR3 (version 2013.0; Academic Medical Center, Amsterdam, The Netherlands). Primer sequences were described in Supplementary Table S2. The most stable reference genes were calculated using geNorm^40^. Transcript levels were normalized to housekeeping genes ratio GAPDH and Cyclophilin (CyP) for MCF-7 and Fibroblasts cells, and the genes ratio HPRT and Actin for HepG2 cells.

#### Western blot

MCF-7 cells were seeded in a 12 wells plate until reaching confluence. Then the cells were incubated with vehicle (0) or fruti at 10, 25 and 50 µM for 24 h (concentrations previously chosen by WST1 assay screening), harvested in iced cold PBS and homogenized in RIPA buffer freshly supplemented with PhosSTOP phosphatase inhibitor (Roche, Mannheim, Germany) and 5 mM EDTA-free protease inhibitor cocktail (Roche, Mannheim, Germany). The samples were centrifuged for 10 min at 4 °C and the supernatant was used for protein measurement by the bicinchonic acid assay. Forty micrograms of protein were electrophoresed on 8–10% sodium dodecyl sulfate–polyacrylamide gel and transferred to a polyvinylidine difluoride (PVDF) membrane by semidry blotting. The membrane was blocked overnight in 5% nonfat milk/TBST followed of overnight incubation with primary antibody in a cold room at 4 °C. The PVDF membrane was washed 3 times with TBST and incubated with secondary antibody with horseradish peroxidase-conjugated goat anti-rabbit or anti-mouse immunoglobulin G antibody (BioRad, California, USA) for 1 h at room temperature. All antibodies were diluted in 1% nonfat milk/TBST. The lists of antibodies and dilutions used were described in Supplementary Table S3. The bands in the membrane were detected using chemiluminescence reagents (100 mM Tris HCl pH 8.5, 1.25 nM luminol 0.2 mM p-coumarin and freshly added 3 mM H_2_O_2_) by ImageQuant LAS 4000 (GE Healthcare Life Sciences, Pittsburgh, USA) and compared with endogenous GAPDH protein.

#### Caspase 3/7 activity assay

As human MCF-7 cells do not express caspase 3 protein^41^, the caspase 3/7 activity assay was performed seeding HepG2 cells in a 96 wells plate. The cells were incubated with vehicle (0) or fruti at 10, 25, 50, 75, 100 and 125 µM for 16, 24, 48 and 72 h to better detect the effect of different fruti concentrations on caspase 3/7 activity over a time-course. Caspase 3/7 activity solution was added according to the manufacture instructions (SensoLyte, AnaSpec EGT Group, Fremont, USA). The fluorescence was monitored at 485–490/520–535 nm on a Clariostar analyzer at 37 °C. Results were expresses as relative fluorescence.

### In vivo experiments

#### Animals

Female Swiss mice (weighing 25-30 g) were housed with 7 animals per cage with free access to food and water and maintained under controlled conditions of humidity, luminosity (12/12 h ligh/dark cicle) and temperature (± 22 °C). All experiments were approved by local Ethics Committee of Animal Experimentation (CEUA/BIO – UFPR; 879) and animals were randomized before the treatments. Experiments were performed in accordance with ARRIVE international guidelines^42^. Efforts were made to avoid animal suffering, and environmental enrichment was used in the cages during all of the experimental protocols.

#### Ehrlich Carcinoma manipulation and groups of treatment

Ascites Ehrlich cells (2 × 10^6^ cells/animal) were maintained by weekly intraperitoneal passage. After cells reach ≥ 98% of viability they were inoculated subcutaneously (2 × 10^6^ cells/animal) in the right pelvic member of mice for SEC development. Animals were orally and daily treated with vehicle (0.1% Tween 20 in distilled water, 10 mg mL^−1^) or fruti 3 mg kg^−1^ for 21 days. This dose of fruti was based on the yield of the ethanolic extract effect, previously prepared by our group^11^. Additionally, a separate positive control group was treated with methotrexate (MTX) 2.5 mg kg^−1^ every 3 days by intraperitoneal route during 21 days. The tumor volume was measured from day 7 until day 21 and was calculated according to Mishra et al.^43^ as $V\left({\mathrm{cm}}^{3}\right)= L \times {W}^{2}\times 0.52$, where *L* is the largest tumor diameter and *W* is the smallest tumor diameter (in centimeters). On day 22, the mice were fasted for 16 h and anesthetized with ketamine (90 mg kg^−1^) and xylazine (10 mg kg^−1^) (Vetnil, São Paulo, Brazil) by intraperitoneal route, and blood, tumor and organs were collected for further analysis.

#### Total RNA isolation and RT-qPCR

The expression of target genes for proliferation, apoptosis, necroptosis and angiogenesis was assessed in tumor samples. RNA isolation and complementary DNA synthesis were performed as described in Sect. 2.3.4. Primers sequences are described in Supplementary Table S4. Relative expression levels were calculated using *Gapdh* as endogenous control gene.

#### Western blot in tumor tissue

Tumor samples were homogenized in protein extraction buffer (10 mM ethylenediaminetetraacetic acid, 100 mM Tris [pH 7.5], and 0.4% protease inhibitor cocktail; Promega, São Paulo, Brazil)^44^. After adding 100 μL of 10% Triton X-100 the samples were cooled for 30 min and centrifuged at 16,000×g for 40 min at 4 °C. Laemmli buffer (50 μL; 0.1% bromophenol blue, 1 M sodium phosphate [pH 7.0], 50% glycerol, 10% sodium dodecyl sulfate [SDS]^45^, and 100 mM dithiothreitol) were added to 200 μL of the supernatant, followed by heating in boiling water for 5 min and then storage at − 20 °C. For electrophoresis, a volume equivalent to 150 μg of protein per sample was added to the 10% SDS–polyacrylamide gel electrophoresis (PAGE) gel, and a molecular weight standard was used (Precision Plus Proteine Standards All Blue, Bio-Rad). Protein separation was performed at 60 V for the first 30 min and 160 V until the end of the run. The transfer protein into nitrocellulose membranes (Hybond ECL, GE Healthcare Life Sciences, São Paulo, Brazil) was performed through wet-transfer method at 120 V per 90 min using a transfer buffer (25 mM Tris [pH 7.6], 192 mM glicine, 20% methanol and 0.02% SDS). The membranes were stained using Ponceau S (sc-301558, Santa Cruz Biotechnologies, Dallas, TX, USA) and then cut. Nonspecific bindings were blocked using basal solution (20 mM Tris [pH 7.6], 137 mM NaCl and 0.025% Tween 20 for all subsequent serial washes) with bovine serum albumin (BSA) 5% for 2 h. After serial washes, the samples were incubated overnight with primary anti-NF-κB antibody (1:300; sc-8008, Santa Cruz Biotechnologies, Dallas, TX, USA) or anti-p-NF-κB, containing phosphorylated Ser 536 (1:300; sc-136548, Santa Cruz Biotechnologies, Dallas, TX, USA), in basal solution plus 3% BSA. Several series washes were performed prior to incubation with anti-mouse secondary antibody (1:5000; sc-516102, Santa Cruz Biotechnologies, Dallas, TX, USA) for 1 h. Protein revelation was performed with chemiluminescent reagent (Western ECL Substrate, Bio-Rad) in a photo-documenter (Amersham Imager 600, GE Healthcare Life Sciences). For evaluation of p-NF-kB, the membrane was stripped, blocked and incubated overnight. The quantitative densitometric analysis was then performed using Scion Image Beta 4.03 software (Scion Corporation, USA)^44^. The band intensities were normalized to GAPDH (1:5000; sc-32233, Santa Cruz Biotechnologies, Dallas, TX, USA).

#### Oxidative stress and inflammatory parameters

To measure oxidative stress parameters, tumor tissue was homogenized in phosphate buffer pH 6.5 (1:10) and centrifuged at 10,000×*g* at 4 °C for 20 min. The homogenate was used to measure reduced glutathione (GSH)^46^ and the supernatant was used to measure reactive oxygen species (ROS)^39,47^.

For inflammatory parameters, tumor tissues were homogenized in PBS and centrifuged at 10,000×*g* at 4 °C for 20 min. The supernatant was used to measure NO according to Green et al.^48^ and cytokines (TNF-α, IL-6, IL-10, IL-4) according to the manufacturer’s instructions (Kits BD Bioscience, Califórnia, USA). The pellet was ressuspended in 0.1% Saline-Triton X and centrifuged at 11,000×*g* at 4 °C for 20 min. The supernatant was used to measure myeloperoxidase (MPO)^49^ and *N*-acetyl-β-D-glucosaminidase (NAG) levels^50^.

#### Tumor histology

Tumor and liver tissue were fixed in ALFAC solution (85% ethanol, 10% formaldehyde, 5% glacial acetic acid) at room temperature for 16 h following the routine dehydration for paraffin embedding. Samples were sectioned at 5 µm thickness and stained with hematoxylin and eosin. Analysis was performed by optical microscopy in 3 or 4 slides per group. The evaluated parameters were apoptosis (Grade 0: absent; Grade I: discrete; Grade II: moderate), inflammatory infiltration (Grade 0: absent; Grade I: discrete; Grade II: moderate; Grade III: high) and coagulative necrosis (Grade 0: absent; Grade I: present in 1–20% of the histological area; Grade II: 21–50%; Grade III: 51–80%; Grade IV: 81–100%). Images were acquired microscopically using Axio Imager Z2 epifluorescence (Carl Zeiss, Germany), equipped with an automated slide scanner from MetaSystems (MetaViewer version. 2.0.100, Germany).

#### Plasmatic and hematologic parameters

Hematological parameters were analyzed through complete hemograms using a BC2800-Vet (BioBrasil, São Paulo, Brazil) automated device. Plasma samples were obtained after blood centrifugation at 1344×*g* for 5 min. The samples were used to determine creatinine, glucose, total protein, albumin, globulin, alanine aminotransferase (ALT) and aspartate aminotransferase (AST) levels, according to commercial kits instructions (Kovalent, São Gonçalo, Brazil). All parameters were analyzed by an automated system (Mindray BS-200, Shenzhen, China).

#### Data analysis

Data were expressed as means ± standard error of mean (S.E.M.). Results were subjected to t-test or one- or two-way analysis of variance (ANOVA), followed by post hoc Newman Keuls test, when applicable. The results were analyzed using GraphPad Prism (v. 5.0, San Diego, CA, USA). The level of significance was set at 95% (*p* < 0.05).

## Supplementary information

Supplementary Information.

## Data Availability

The datasets generated during and/or analyzed during the current study are available from the corresponding author on reasonable request.
