# Supplementary material for: Fruticuline A, a chemically-defined diterpene, exerts antineoplastic effects in vitro and in vivo by multiple mechanisms
Source: Sci Rep. 2020 Oct 5;10:16477. doi: 10.1038/s41598-020-73432-2 (PMC7536426; doi:10.1038/s41598-020-73432-2)
Supplement: Supplementary file 1 — Supplementary Information. [file 41598_2020_73432_MOESM1_ESM.docx]

**Fruticuline A, a chemically-defined diterpene, exerts antineoplastic effects *in vitro* and *in vivo* by multiple mechanisms**

Claudia Rita Corso^1,2^, Maria Carolina Stipp^1^, Débora Rasec Radulski^1^, Marihá Mariott^3^, Luiza Mota da Silva^3^, Edneia Amancio de Souza Ramos^4^, Giseli Klassen^4^, José Ederaldo Queiroz Telles^5^, Cristhian Santos Oliveira^6^, Maria Élida Alves Stefanello^6^, Arthur Verhoeven^7^, Ronald Oude Elferink^7^, Alexandra Acco^1*^

^1^ Pharmacology Department, Federal University of Parana, Curitiba, PR, Brazil

^2^ Instituto de Pesquisa Pelé Pequeno Príncipe, Faculdades Pequeno Príncipe, Curitiba, PR, Brazil

^3^ Postgraduate Program in Pharmaceutical Sciences, University Vale of Itajaí, Itajaí, SC, Brazil

^4^ Pathology Department, Federal University of Parana, Curitiba, PR, Brazil

^5^ Medical Pathology Department, Federal University of Parana, Curitiba, PR, Brazil

^6^ Chemistry Department, Federal University of Parana, Curitiba, PR, Brazil

^7^ Academic Medical Center, Tytgat Institute for Liver and Intestinal Research, Amsterdam, The Netherlands

* Corresponding author

Department of Pharmacology, Biological Sciences Sector, Federal University of Parana – UFPR

PO Box 19031, Curitiba, PR, 81531-980, Brazil.

Tel.: +55 41 3361-1742; Fax: +55 41 3366-2042

e-mail: aleacco@ufpr.br

# Supplementary Material

**
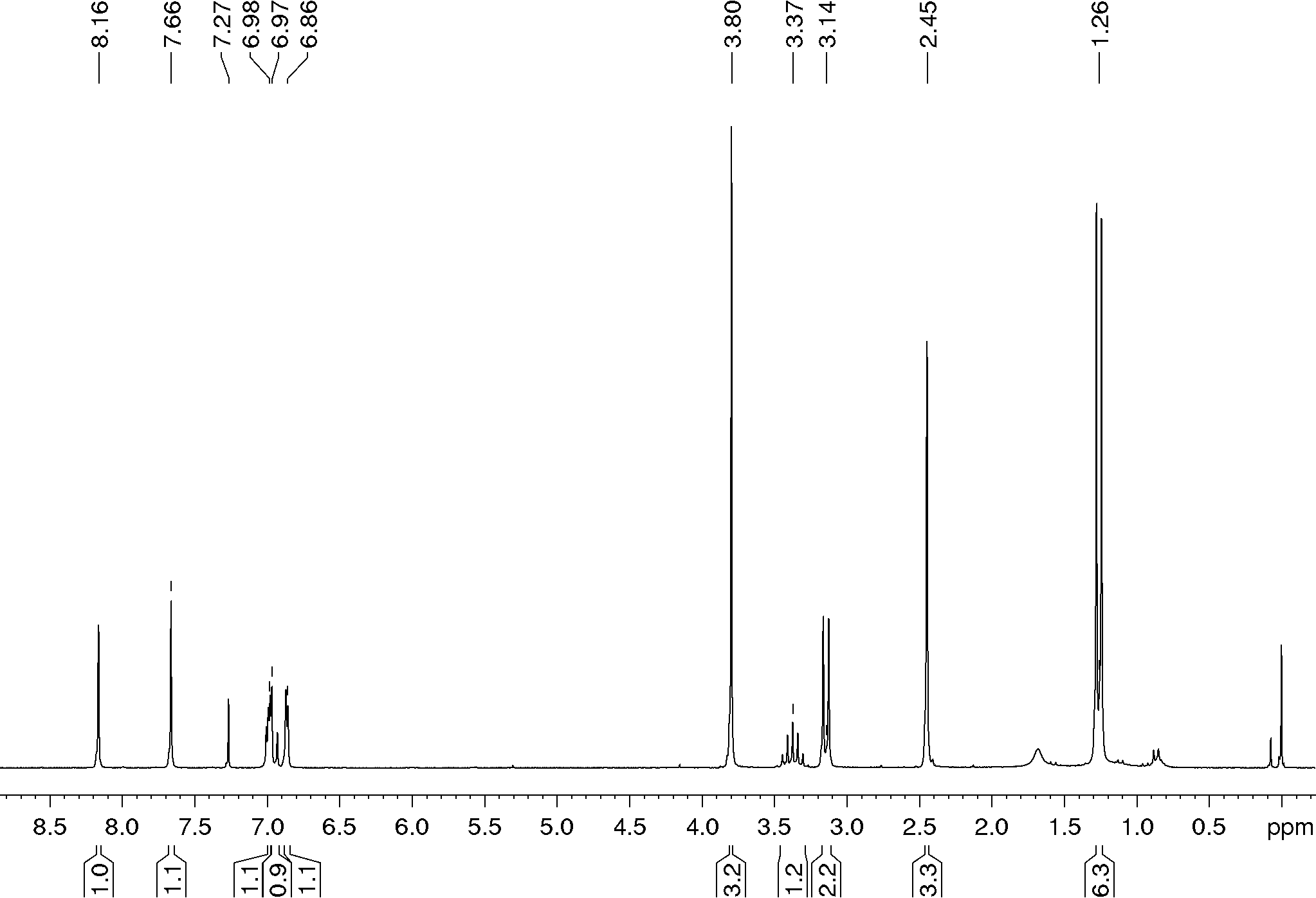
**

**Supplementary Figure S1.** NMR ^1^H spectrum of fruticuline A (CDCl_3_, 200 MHz).


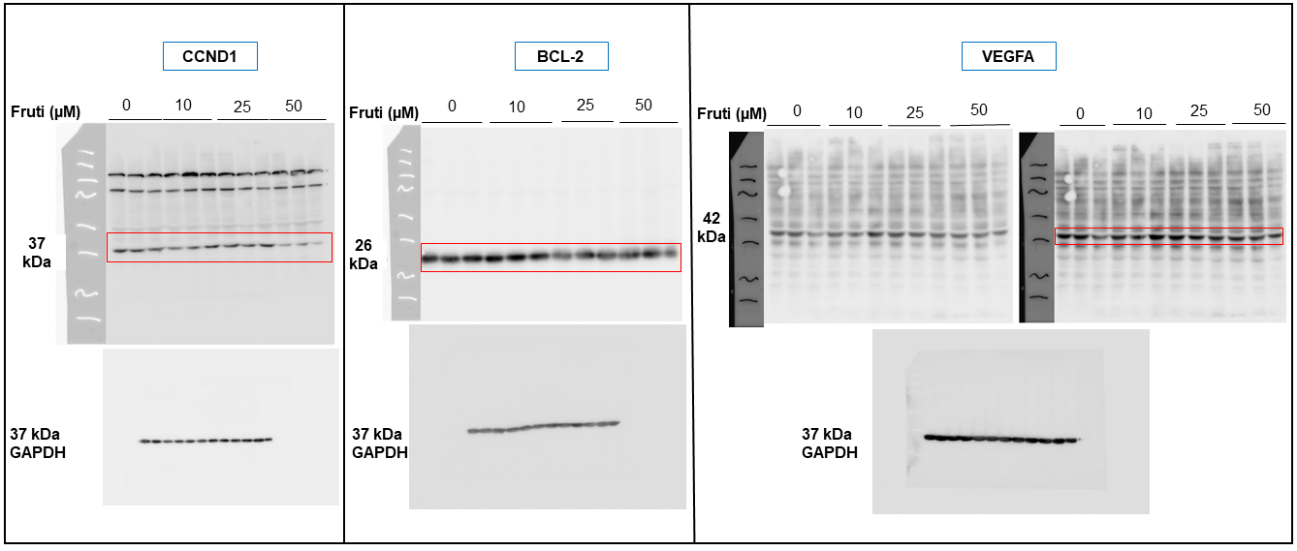


**Supplementary Figure S2.** Full size blot images of CCND1, BCL-2 and VEGFA in MCF-7 cells. For VEGFA cells blots were submitted to two high-contrast to improve bands visibility. Cells were incubated with vehicle (0) or fruti 10, 25 and 50 µM for 24 h.

**Supplementary Figure S3.** WST-1 viability cells assay in the presence and absence of 50 µM Necrostatin in HepG2 cells. Cells were incubated with vehicle (0) or fruti at 15.2, 31.2, 62.5, 125 and 250 µM for 24 h. Results are expressed as mean ± S.E.M. (n= 3) and were analyzed by one-way ANOVA followed by Newman Keuls post hoc test. ^#^ and * *p*<0.05 when compared to vehicle group (0 µM) in absence of Necrostatin-1 (- Nec-1) and fruti concentrations in the absence of Nec-1, respectively.

**Supplementary Figure S4.** NF-κB1 gene expression in HepG2 cells. Cells were incubated with vehicle or fruti at 10, 25 and 50 µM for 24 h. Results are expressed as mean ± S.E.M. (n= 3) and were analyzed by one-away ANOVA followed by Newman Keuls post hoc test. ***** *p*<0.05 when compared to vehicle group.


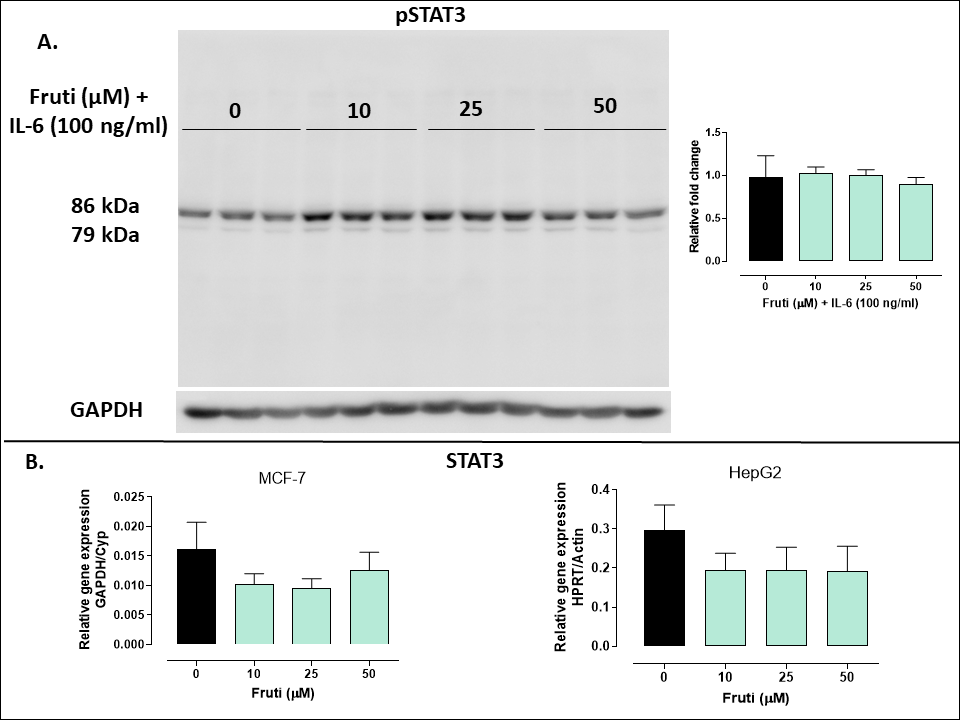


**Supplementary Figure S5.** Protein level of pSTAT3 (Panel **A**) after stimulation of IL- 6 (100 ng ml^-1^) and *STAT3* gene expression in MCF-7 and HepG2 cells (Panel **B**). Cells were incubated with vehicle or fruti at 10, 25 and 50 µM for 24 h. Results are expressed as mean ± S.E.M. (n= 3-6) and were analyzed by one-away ANOVA followed by Newman Keuls post hoc test.


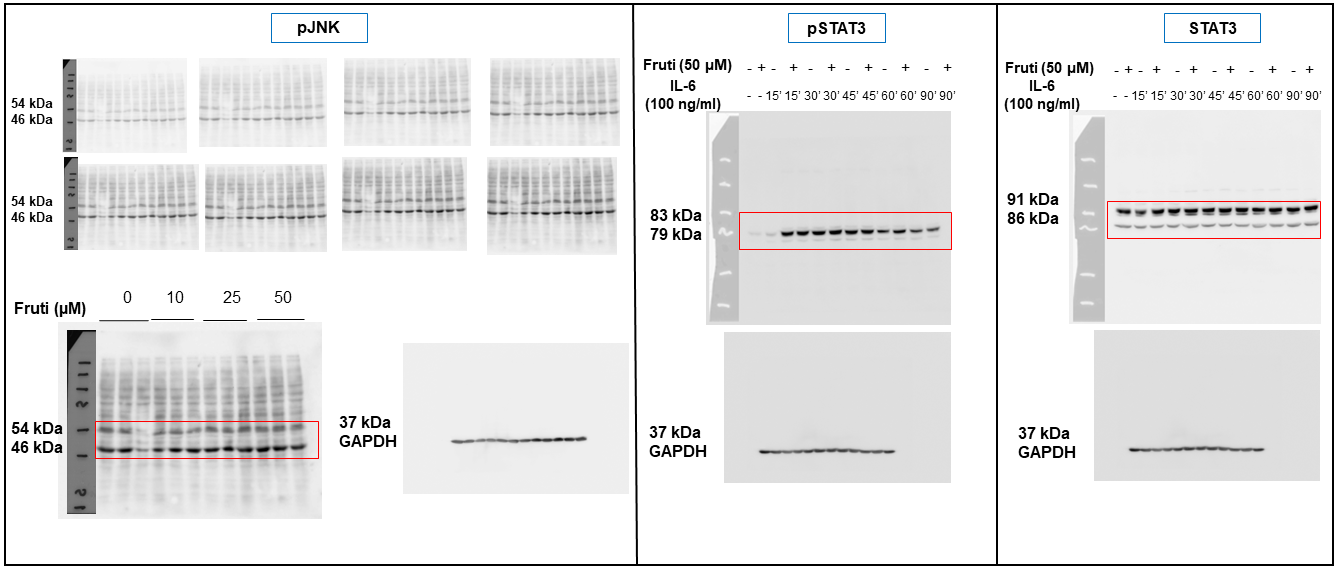


**Supplementary Figure S6.** Full size blot images of pJNK, pSTAT3 and STAT3 in MCF-7 cells. For pJNK cells blots were submitted to multiple high-contrast to improve bands visibility. For pJNK cells were incubated with vehicle (0) or fruti at 10, 25 and 50 µM for 24 h. For pSTAT3 and STAT3 analysis, cells were incubated with vehicle (0) or fruti at 50 µM in the presence or absence of IL-6 (100 ng ml^-1^) for 15 until 90 min.


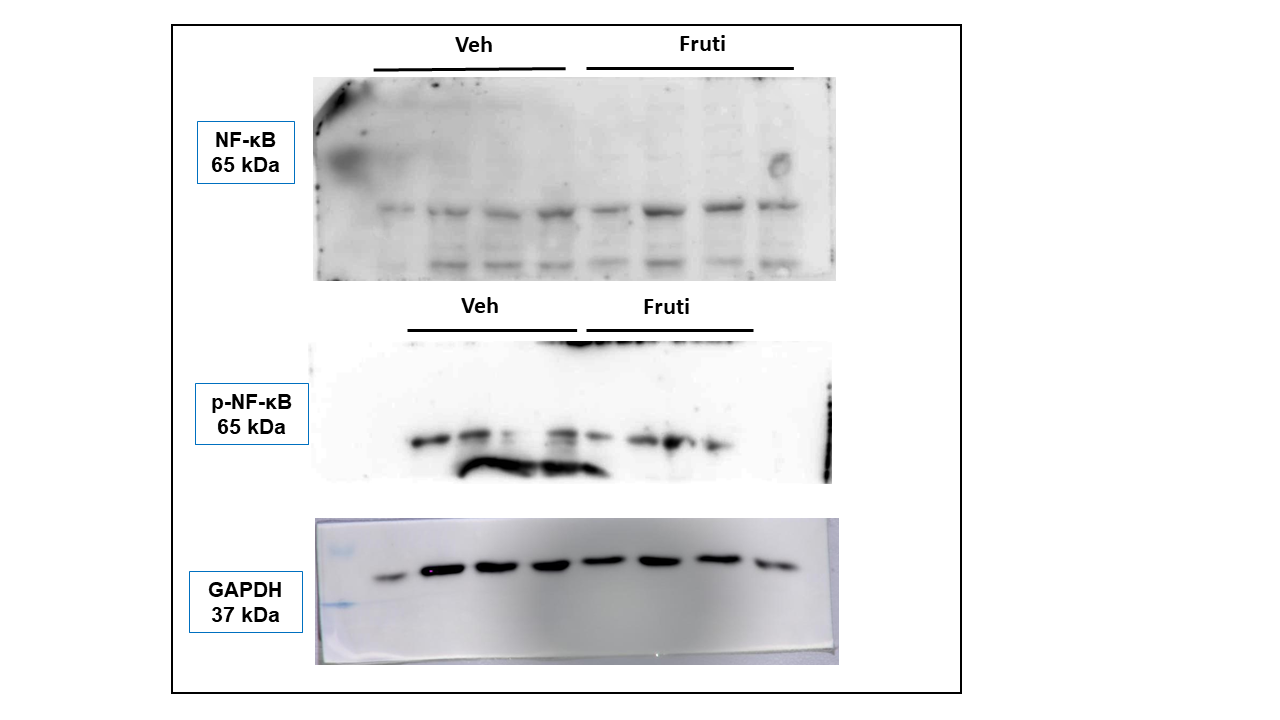


**Supplementary Figure S7.** Full size blot images of NF-κB and p-NF-κB in tumor tissue. For p-NF-κB blots were submitted to multiple high-contrasts to improve bands visibility. Animals were treated with fruti (3 mg kg^-1^, p.o.) or vehicle (Veh, 0.1% tween 20 in distilled water, 10 mL kg^-1^, p.o.) for 21 days.


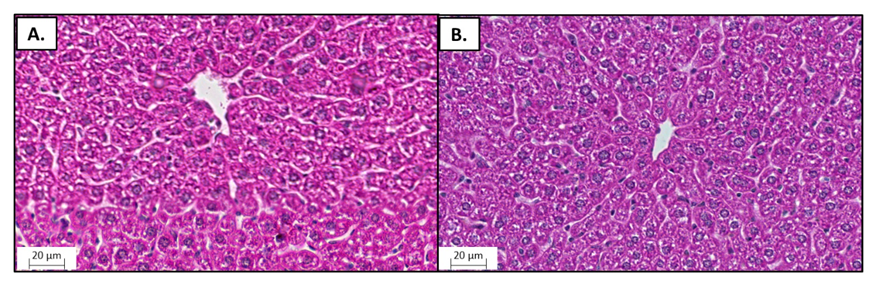


**Supplementary Figure S8.** Liver histology in Ehrlich tumor bearing mice. Animals were treated with vehicle p.o. (Panel **A**) or fruti 3 mg kg^-1^ p.o (Panel **B**) for 21 days. Samples were observed in optical microscope at 20 x (scale bar 20 µm).

**Supplementary Table S1.** Plasmatic parameters in healthy and tumor-bearing mice treated with vehicle, fruti or MTX during 21 days.

|  | **Experimental groups** | | | |
| --- | --- | --- | --- | --- |
| **Parameters** | **Naive** | **Veh** | **Fruti** | **MTX** |
| Glucose (mg dL^-1^) | 122.08 ± 12.17 | 133.02 ± 7.11 | 154.04 ± 15.50 | 119.08 ± 24.69 |
| Creatinine (mg dL^-1^) | 0.68 ± 0.06 | 0.73 ± 0.05 | 0.72 ± 0.04 | 0.63 ± 0.03 |
| AST (U L^-1^) | 86.89 ± 8.72 | 272.02 ± 16.79^#^ | 280.09 ± 28.57^#^ | 276.09 ± 42.00^#^ |
| ALT (U L^-1^) | 65.02 ± 5.54 | 65.94 ± 6.03 | 41.70 ± 2.53*^,#^ | 52.57 ± 5.71 |
| Total protein (g dL^-1^) | 5.91 ± 0.16 | 6.56 ± 0.10^#^ | 5.81 ± 0.13* | 4.30 ± 0.24*^,#^ |
| Globulin (g dL^-1^) | 2.80 ± 0.07 | 2.76 ± 0.11 | 3.32 ± 0.15 | 1.54 ± 0.38*^,#^ |
| Albumin (g dL^-1^) | 3.09 ± 0.08 | 3.92 ± 0.21^#^ | 3.32 ± 0.15* | 2.78 ± 0.24* |
| Leukocytes (^x^10^3^ µL^-1^) | 5.64 ± 0.79 | 5.16 ± 0.44 | 7.00 ± 0.61 | 5.83 ± 1.17 |
| Lymphocytes (^x^10^3^ µL^-1^) | 3.90 ± 0.60 | 3.71 ± 0.27 | 5.37 ± 0.57 | 3.73 ± 0.84 |
| Monocytes (^x^10^3^ µL^-1^) | 0.20 ± 0.04 | 0.15 ± 0.02 | 0.28 ± 0.02* | 0.36 ± 0.07*^,#^ |
| Erythrocytes (^x^10^6^ µL^-1^) | 9.85 ± 0.10 | 9.51 ± 0.17 | 9.79 ± 0.27 | 8.35 ± 0.32*^,#^ |
| Hemoglobin (g dL^-1^) | 13.28 ± 0.19 | 12.52 ± 0.27 | 13.45 ± 0.38 | 11.58 ± 0.38*^,#^ |
| Hematocrit (%) | 44.58 ± 0.99 | 41.46 ± 0.83 | 42.89 ± 1.01 | 35.13 ± 1.38*^,#^ |

Results were expressed as mean ± S.E.M. and were analyzed by one-way ANOVA followed by Newman-Keuls post hoc test (n=5-8). * and ^#^ indicated *p*<0.05 when compared to vehicle and naive group, respectively.

**Supplementary Table S2.** Gene sequences of real-time quantitative PCR primers for human cells.

| Target | Forward primer (5’**🡪**3’) | Reverse primer (5’**🡪**3’) |
| --- | --- | --- |
| ***VEGFA*** | ATCTGCATGGTGATGTTGGA | GGGCAGAATCATCACGAAGT |
| ***CCND1*** | CCGTCCATGCGGAAGATC | GAAGACCTCCTCCTCGCACT |
| ***BCL-2*** | GATTGTGGCCTTCTTTGAG | CAAACTGAGCAGAGTCTTC |
| ***NF-κB1*** | TACTCTGGCGCAGAAATTAGGTC | CTGTCTCGGAGCTCGTCTATTTG |
| ***STAT3*** | AAAGTGCCTTTGTGGTGGAG | TGTGTTTGTGCCCAGAATGT |
| ***RIPK1*** | GGCATTGAAGAAAAATTTAGGC | TCACAACTGCATTTTCGTTTG |
| ***TNF-α*** | CCTGCTGCACTTTGGAGTGA | GAGGGTTTGCTACAACATGGG |
| ***GAPDH*** | AGGTCGGTGTGAACGGATTTG | TGTAGACCATGTAGTTGAGGTCA |
| ***CyP*** | ACGGCGAGCCCTTGG | TTTCTGCTGTCTTTGGGACCT |
| ***ACTIN*** | GAGCACAGAGCCTCGCCTTT | TCATCATCCATGGTGAGCTGG |
| ***HPRT*** | TGACCTTGATTTATTTATTTTGCATACC | CGAGCAAGACGTTCAGTCCT |

**Supplementary Table S3.** List of immunoblotting antibodies for human cells.

| Target Protein | Company | Catalog No. | Source | Isotype | Dilution |
| --- | --- | --- | --- | --- | --- |
| **VEGFA** | Santa Cruz | (C-1) sc-7269 | mouse | IgG | 1:200 |
| **CCND1** | Santa Cruz | (A-12) sc-8396 | mouse | IgG | 1:200 |
| **BCL-2** | Santa Cruz | (100) sc-509 | mouse | IgG | 1:200 |
| **pSTAT3** | Cell signaling | 9145 | rabbit | IgG | 1:2000 |
| **STAT3** | Santa Cruz | (F-2) sc-8019 | mouse | IgG | 1:200 |
| **pJNK** | Cell signaling | 4668 | rabbit | IgG | 1:1000 |
| **GAPDH** | Cell signaling | 5174 | rabbit | IgG | 1:2000 |

**Supplementary Table S4**. Gene sequences of real-time quantitative PCR primers for mice tissue.

| Target | Forward primer (5’**🡪**3’) | Reverse primer (5’**🡪**3’) |
| --- | --- | --- |
| ***Vegfa*** | ACTGGACCCTGGCTTTACTGCT | TGATCCGCATGATCTGCATGGTG |
| ***Ccnd1*** | AGAAGTGCGAAGAGGAG | GGATAGAGTTGTCAGTGTAGAT |
| ***Bcl-2*** | CACTTGCCACTGTAGAGA | GCTTCACTGCCTCCTT |
| ***Rela*** | ACCTGGAGCAAGCCATTAGC | GAGGCGCACTGCATTC |
| ***IkBa*** | GCTACCCGAGAGCGAGGAT | GCCTCCAAACACACAGTCATCAT |
| ***Gapdh*** | GGTGAAGCAGGCATCT | TGTTGAAGTCGCAGGAG |
